# Glyco-Engineered Anti-Human Programmed Death-Ligand 1 Antibody Mediates Stronger CD8 T Cell Activation Than Its Normal Glycosylated and Non-Glycosylated Counterparts

**DOI:** 10.3389/fimmu.2018.01614

**Published:** 2018-07-16

**Authors:** Christoph Goletz, Timo Lischke, Ulf Harnack, Phillip Schiele, Antje Danielczyk, Johanna Rühmann, Steffen Goletz

**Affiliations:** Glycotope GmbH, Berlin, Germany

**Keywords:** anti-programmed death-ligand 1, antibody, Fc part, glyco-engineering, defucosylation, T cell activation

## Abstract

The programmed death 1 (PD-1)/programmed death-ligand 1 (PD-L1) axis plays a central role in suppression of anti-tumor immunity. Blocking the axis by targeting PD-L1 with monoclonal antibodies is an effective and already clinically approved approach to treat cancer patients. Glyco-engineering technology can be used to optimize different properties of monoclonal antibodies, for example, binding to FcγRs. We generated two glycosylation variants of the same anti-PD-L1 antibody: one bearing core fucosylated N-glycans in its Fc part (92%) and its de-fucosylated counterpart (4%). The two glycosylation variants were compared to a non-glycosylated commercially available anti-PD-L1 antibody in various assays. No differences were observed regarding binding to PD-L1 and blocking of this interaction with its counter receptors PD-1 or CD80. The de-fucosylated anti-PD-L1 antibody showed increased FcγRIIIa binding resulting in enhanced antibody dependent cellular cytotoxicity (ADCC) activity against PD-L1^+^ cancer cells compared to the “normal”-glycosylated variant. Both glycosylation variants showed no antibody-mediated lysis of B cells and monocytes. The non-glycosylated reference antibody showed no FcγRIIIa engagement and no ADCC activity. Using mixed leukocyte reaction it was observed that the de-fucosylated anti-PD-L1 antibody induced the strongest CD8 T cell activation determined by expression of activation markers, proliferation, and cytotoxicity against cancer cells. The systematic comparison of anti-PD-L1 antibody glycosylation variants with different Fc-mediated potencies demonstrates that our glyco-optimization approach has the potential to enhance CD8 T cell-mediated anti-tumor activity which may improve the therapeutic benefit of anti-PD-L1 antibodies.

## Introduction

The programmed death 1 (PD-1)/programmed death-ligand 1 (PD-L1) axis has a central role in suppression of anti-tumor immunity (1). PD-L1 (also known as CD274 or B7-H1), a B7 family member, is a transmembrane protein broadly expressed not only in hematopoietic cells, such as B and T lymphocytes and macrophages but also on nonhematopoietic cells (2, 3). Typically, PD-L1 expression is induced during inflammatory conditions, e.g., in presence of interferon gamma (IFN-γ) (2, 3). PD-1, the receptor of PD-L1, is a member of the CD28 family expressed on activated T cells (4). Engagement of PD-1 on T cells by PD-L1 leads to their functional suppression evident by decreased cytokine production and proliferation (3). The tissue expression of PD-L1 is essentially involved in mediating peripheral T cell tolerance (5), whereas PD-L1 expression on APC is decisive for regulating T cell immune responses in lymphoid tissues (6). In addition to its function as a ligand for PD-1, PD-L1 can additionally bind to CD80 also resulting in inhibition of T cell activation (7).

Tumor-infiltrating T lymphocytes (TILs) express high levels of PD-1 (8). It has also been shown that TILs produce elevated levels of IFN-γ, which results in upregulation of PD-L1 on the tumor cell surface (9). Thereby, tumor tissues use PD-L1 expression as an escape mechanism from immune surveillance by dampening T cell activity (10). Unsurprisingly, PD-L1 is widely expressed on a variety of different tumor tissues (10). The role in immune escape is also emphasized by the finding that PD-L1 expression on tumor cells is often associated with poor prognosis observed for several cancer types, e.g., breast (11) and bladder carcinoma (12).

The therapeutic potential of PD-1/PD-L1 blockade has been demonstrated in multiple cancer mouse models (13, 14) and in human clinical trials (15–19). Two anti-PD-1 antibodies were approved for different indications in the last 4 years: nivolumab (Bristol-Myers Squibb) and pembrolizumab (Merck & Co.). Similarly, the following anti-PD-L1 antibodies have recently been approved for specific indications: atezolizumab (MPDL3280A, Genentech), avelumab (EMD Serono and Pfizer), and durvalumab (AstraZeneca). Summarized, these anti-PD-1 and anti-PD-L1 antibodies improve the outcome of anti-cancer immune therapies as well as anti-viral therapies (1). Mechanistically, it has been shown that the blockade of co-inhibitory immune checkpoint molecules like PD-1 and PD-L1 increases the T-cell-specific immune response that turns the immune system against the tumor (1). However, although therapies blocking the PD-1/PD-L1 axis show impressive long lasting efficacies, curative responses are only observed in certain carcinomas and in a subset of patients (1).

The role of the Fc part of anti-PD-1 and anti-PD-L1 antibodies in cancer therapy has been discussed controversially. Antibodies with Fc-mediated effector functions may mediate antibody dependent cellular cytotoxicity (ADCC) against PD-1 and PD-L1 expressing TILs, and thus may significantly change the effects of therapy (20). In the human system, ADCC is mediated *via* the activating FcγRIIIa which is prominently expressed on NK cells (21, 22). All approved anti-PD-1 antibodies are of the human IgG4 isotype (15, 16) having low affinity to FcγRIIIa (22) to avoid Fc-mediated cytotoxic effects. Two of the currently approved anti-PD-L1 antibodies are of the human IgG1 isotype but have modifications in the Fc region to eliminate FcγR binding and resulting effector functions (14, 23). In contrast, one approved PD-L1-targeting antibody (avelumab) is a fully functional human IgG1 designed to mediate ADCC (24). Interestingly, it has recently been shown in a murine tumor model that anti-PD-1/PD-L1 antibodies differ in their FcγR requirements for optimal activity: FcγR engagement compromises the anti-tumor activity of anti-PD-1 antibodies, but binding to activating FcγR augments the anti-tumor effects of anti-PD-L1 antibodies (13). Therefore, it was suggested that engineering of the Fc part for enhanced binding to activating FcγRs might be a strategy to optimize the anti-tumor activity of anti-PD-L1 antibodies (13, 25). Human IgG antibodies typically have two conserved N-linked oligosaccharides, each of which is attached to the asparagine on position 297 of the heavy chain (26). Removal of the total N-glycans results in loss of FcγR binding capacity of the antibody (27), whereas removal only of the core fucose from the N-glycans typically leads to increased affinity for FcγRIIIa (21). Thus, we hypothesized that glyco-optimization of anti-PD-L1 antibodies by reduction of the fucosylation degree might be beneficial and result in enhanced therapeutic responses.

Using the human expression platform GlycoExpress we generated an anti-PD-L1 hIgG1 with “normal” N-glycosylation in its Fc region. In addition, we generated a glyco-engineered variant of the same anti-PD-L1 hIgG1 with reduced core fucosylation. We compared both variants to a non-glycosylated reference antibody with identical antigen binding to PD-L1 but different affinities for FcγRIIIa which was highest for the glyco-engineered anti-PD-L1. Enhanced binding to FcγRIIIa was reflected by an increased capacity to mediate ADCC against PD-L1^+^ cancer cells. However, the normal glycosylated as well as the glyco-engineered anti-PD-L1 antibody mediated no ADCC against PD-L1 expressing B cells and monocytes. Remarkably, the glyco-engineered anti-PD-L1 induced enhanced CD8 T cell activation in a mixed leukocyte reaction (MLR) determined by expression of activation markers, proliferation, and cytotoxicity against cancer cells, suggesting an improved therapeutic benefit.

## Materials and Methods

### Construction and Production of Anti-PD-L1 Variants

The variable region for the glycosylated anti-PD-L1 variants is based on the sequence of atezolizumab (Genentech) (23). The antibody sequences of the variable heavy (VH) and light (VL) region were cloned into expression vectors containing sequences for the human constant domains of the IgG1 κ light chain and heavy chain (Glycotope), respectively. Both plasmids were co-transfected in two GlycoExpress cell lines (Glycotope) (28) characterized by normal and reduced core fucosylation followed by selection and gene amplification by increasing concentrations of methotrexate (Sigma-Aldrich, #M8407) and puromycine (Clontech, #631306). High producing cell clones isolated from semisolid matrix medium by the ClonePix system (Molecular Devices) were expanded and used for production of supernatants in spinner culture flasks or 2 l perfusion bioreactors. Antibodies were purified using protein A chromatography on MabSelect Sure (GE-Healthcare, #29049104) to a monomer content >98% and showed no obvious endotoxin-contamination as tested *via* LAL endotoxin assay (GenScript).

Atezolizumab as a reference material was purchased from Genentech (PZN#11306050).

### N-Glycan Analytics

Antibodies were denatured by RapiGest™ SF (Waters Inc., #186002123) and tris-(2-carboxyethyl)phosphine (120 min, 95°C). *N*-acetylglucosamine-linked oligosaccharides were released by enzymatic digestion with Rapid PNGase F (10 min, 55°C) (Waters Inc., #186007990) followed by fluorescence tagging with RapiFluor-MS reagent (Waters Inc., #186007989) in dimethylformamide for 5 min at room temperature (RT). For clean-up of tagged glycans a μElution Plate (HILIC SPE, Waters Inc., #186002780) was used. Labeled N-glycans were analyzed by LC–MS employing a HILIC phase (Acquity UPLC BEH GLYCAN 1.7 μ, 2.1 × 150 mm; Waters Inc., #186004742) with an I-class UPLC (Waters Inc.) coupled to a high resolution QTOF mass spectrometer (Impact HD; Bruker Daltonik). Labeled N-glycans are separated using a gradient from 22% B to 44% B within 82 min (mobile phase A: acetonitrile; mobile phase B: 100 mM ammonium formate in H2O, pH4.4). RapiFluor-MS tagged N-glycans were detected with a fluorescence detector at 265 nm excitation wavelength and 425 nm emission wavelength. Fluorescence signals were employed for glycan quantification. Identification of glycan structures was performed by MS and a series of MS/MS experiments using DataAnalysis Software 4.4 (Bruker Daltonik).

### FcγRIIIa Binding

Binding to hFcγRIIIa was assessed using an assay based on the Alpha technology (Perkin Elmer). In brief, serial dilution of test antibody was mixed with 0.5 µg/ml recombinant His-tagged hFcγRIIIa-158-V (Glycotope) for 30 min at RT using a shaking device. Afterward, 20 µg/ml rabbit-anti-mouse antibody coated acceptor beads and 20 µg/ml Ni-chelate donor beads (both PerkinElmer, #AS101M) were added and incubated for 1 h at RT protected from light. All reagents were diluted in AlphaLisa Universal Buffer (PerkinElmer, #AL001F). Binding of rabbit-anti-mouse antibody coated acceptor beads to FcγRIIIa coupled to donor beads was inhibited by competitive binding of test antibody to FcγRIIIa coupled to donor beads. The disturbed interaction between donor and acceptor beads was assessed by detection of the decreasing chemiluminescence signal quantified by measurement at 520–620 nm in EnSpire 2300 multilabel reader (PerkinElmer).

### Enzyme-Linked Immunosorbent Assays (ELISA)

For the PD-L1 antigen ELISA, plates (Thermo Fisher Scientific, #442404) were coated overnight at 4°C with 0.75 µg/ml hPD-L1 (SinoBiological, #10084-H08H) dissolved in PBS. After plates were washed with PBS + 0.05% Tween-20 (Carl Roth, #9172.2), blocked for 2 h at RT with PBS + 2% BSA (Carl Roth, #8076.3), and washed again, serial dilutions of test antibodies were added and incubated for 1 h at RT. After washing, binding was detected using an anti-hIgG Fc-POD (Jackson ImmunoResearch, #109-035-098) diluted in PBS + 1% BSA which was developed with TMB (tebu-bio/BPS Bioscience, #TMB100-0500) and stopped by 1.25 M H_2_SO_4_. Fluorescence was measured at 450–620 nm at an Infinite F200 plate reader (TECAN).

For the PD-1 and CD80 blockade ELISA hPD-L1-Fc (tebu-bio/BPS Bioscience, #71104) was used for coating. Test antibodies were incubated in presence of 1 µg/ml PD-1-Biotin (tebu-bio/BPS Bioscience, #71109) or 0.5 µg/ml CD80-Biotin (tebu-bio/BPS Bioscience, #71114). Binding was detected using streptavidin-POD (Jackson ImmunoResearch, #016-030-084) 1:15,000-diluted in PBS + 1% BSA.

### Cell Culture, Cell Lines, and Primary Material

The cell lines 5637 (DSMZ, #ACC-35), DU-145 (DSMZ, #ACC-261), KHYG-1 (DSMZ, #ACC-725), Ramos (DSMZ, #ACC-603), and ZR-75-1 (ATCC, #CRL-1500) were obtained from the DSMZ or the ATCC. All cell cultures were free of *Mycoplasma* and maintained in RPMI1640 medium (Biochrom, #F1215) supplemented with 10% FCS (Biochrom, #S0115) and 1% l-glutamine (Biochrom, #K0283). KHYG-1 medium additionally contained 10 ng/ml IL-2 (PeproTech, #200-02). KHYG-1 cells were stably transfected with hFcγRIIIa 158V (KHYG-1-CD16aV), cloned, and maintained in medium containing 25 nmol/l methotrexate (Sigma-Aldrich, #M8407).

Human PBMCs were isolated from leukapheresis products (Charité University Hospital Berlin) or commercially available buffy coats (DRK Berlin) of healthy donors *via* density gradient centrifugation using Biocoll Separating Solution (Biochrom, #L6113). In case of leukapheresis products, isolated PBMCs were stored in AIM-V medium (Life Technologies, #31035025) supplemented with 75% FCS (Biochrom, #S0115) and 10% DMSO (Sigma-Aldrich, #D2650) in liquid nitrogen for future usage. All leukapheresis donors were informed of the purpose of the donation and gave their written consent prior to the study.

PBMC subpopulations were isolated using magnetic cell separation: monocytes (Invitrogen, #11350D or Miltenyi Biotec, #130-096-537), B cells (Invitrogen, #11351D), and T cells (Invitrogen, #11344D).

### Cytotoxicity Assays

The ADCC assay against DU-145 was performed as europium release assay using KHYG-1-CD16aV as effector cells. Briefly, targets were loaded with europium by electroporation (Nucleofector, Lonza) and incubated with a dilution series of test antibody and KHYG-1-CD16aV (effector:target ratio, 10:1) for 5 h at 37°C. Europium release was quantified by time-resolved fluorescence (Infinite F200, Tecan). Maximum release was measured after incubation of target cells with Triton X-100 (Sigma-Aldrich, #T-9284) and spontaneous release was obtained from samples containing only target cells. Percentage of specific lysis was calculated as follows: (experimental − spontaneous)/(maximum − spontaneous) × 100.

Additionally, B cells or monocytes were used as target cells and therefore stained for 20 min at 37°C with 0.2 µM calcein (Sigma-Aldrich, #C1359). Thereafter, calcein-labeled B cells or monocytes were subjected to a cytotoxicity *in vitro* assay with KHYG-1-CD16aV effectors (effector:target ratio, 10:1) in presence of test antibody and RPMI1640 medium supplemented with 5% FCS for 4 h at 37°C. Anti-CD20 antibody obinutuzumab (Roche, PZN#10048686) or staurosporine (Sigma-Aldrich, #S6942) served as positive controls. After 4 h at 37°C, cells were stained with 7-AAD (Sigma-Aldrich, #A9400) and analyzed by flow cytometry. Cytotoxicity against B cells or monocytes was given as relative frequency of 7-AAD^+^ of calcein^+^ target cells.

### Mixed Leukocyte Reaction

For generation of monocyte-derived dendritic cells (moDCs), monocytes were cultured in MEM Alpha Medium (Thermo Fisher Scientific, #22571) supplemented with 20% FCS (Biochrom, #S0115) and 10% conditioned medium (supernatant of 5637 cell culture), 250 U/ml GM-CSF (Gentaur Pharmaceuticals, #04-RHUGM-CSF), and 500 U/ml IL-4 (PeproTech, #200-04) for 7 days. After harvesting, moDCs were cultured in 96-well flat bottom plate (TPP, #92696) together with allogenic T cells at a ratio of 1:10 in the presence of 1 µg/ml of test antibodies in RPMI1640 medium (Biochrom, #F1215) supplemented with 10% FCS (Biochrom, #S0115) and 1% l-glutamine (Biochrom, #K0283). Either, supernatants were harvested on day 2 for a hIL-2 ELISA (eBioscience/Affimetryx, #88-7025-77) or cells were harvested on day 5 and subjected to flow cytometric analysis or to a cytotoxicity assay.

Surface staining for flow cytometry was performed with a variety of monoclonal antibodies coupled to fluorophores: αCD3-APC-H7 (clone: SK7, #560176), αCD3-BV711 (clone: UCHT-1, #563724), αCD8-BV605 (clone: SK1, #564115), and αCD8-FITC (clone: RPA-T8, #555366) were purchased from Becton Dickinson Biosciences. αCD3-PerCPVio700 (clone: REA613, #130-109-465) was purchased from Miltenyi Biotec. αCD4-FITC (clone: RPA-T4, #300538), αCD25-PE (clone: BC96, #302606), αCD56-BV711 (clone: 5.1H11, #362542), and αCD137 (4-1BB)-AF647 (clone: 4B4-1, #309824) were purchased from BioLegend. Dead cells were excluded by 7-AAD (CalBiochem, #129935) or DAPI (4-,6-diamidino-2-phenylindole, Merck, Germany, #124653 staining for analysis. Surface stained cells were analyzed with a FACS Canto II (Becton Dickinson Biosciences) or Attune NxT (Thermo Fisher Scientific) flow cytometer. Data were evaluated using the FlowJo 10 software (Treestar). Gates for positive markers were set according to isotype controls.

To analyze proliferation, allogenic T cells were labeled in some cases with CFSE (5-6-CFDA, Invitrogen, #C1157) before the MLR assay, according to standard protocols.

Cytotoxicity of T cells was determined in an europium release assay as described above using harvested T cells as effectors and ZR-75-1 as targets (effector:target ratio, 10:1–100:1) in the absence or presence of 13.5 or 40.4 µg/ml of a bispecific antibody binding to a tumor antigen on ZR-75-1 and to CD3 on T cells (Glycotope). The effector:target ratio and the concentration of the bispecific antibody used within each experiment was consistent.

### Calculation of Relative Potencies and Statistical Analysis

Calculation of relative potencies and statistical analyses was performed with Prism 5.04 software (GraphPad Software Inc.). Relative potency of hFcγRIIIa binding was determined by calculating the half maximal effective concentration (EC50) for the test and reference antibody using a sigmoidal dose-response model with variable slope constraining the values for bottom, top, and Hill slope. Relative potency was calculated as follows: EC50 of reference antibody divided by EC50 of test antibody.

Statistical analysis was performed using One-Way ANOVA and Bonferroni post test. A *p* value of <0.05 was considered significant (*p < 0.05; **p < 0.01; ***p < 0.001; and ****p < 0.0001; ns = not significant). In case of One-Way ANOVA with repeated measures, Gaussian distribution was confirmed using the Kolmogorov–Smirnov test.

## Results

### Generation of a Normal Glycosylated and a Glyco-Engineered Anti-PD-L1 Antibody With Different FcγRIIIa Binding Capacities

Two of the approved anti-PD-L1 antibodies lack Fc-mediated effector functions due to modifications in the Fc region in order to avoid potential ADCC against PD-L1-expressing effector cells (14, 23). However, it was recently shown that the Fc region contributes to the efficacy of anti-PD-L1 antibodies in the way that isotypes with higher affinity to activating FcγR mediate increased anti-tumor activity (13) providing us the rationale to investigate if glyco-engineering of anti-PD-L1 antibodies might result in further improvement.

Atezolizumab is a humanized IgG1 antibody which lacks the N-glycosylation site in its Fc region by changing an aspartic acid into alanine at amino acid position 298 (amino acid position 297 according to EU nomenclature) in the heavy chain leading to minimized binding to FcγRs (23) (atezolizumab is referred to as non-glycosylated anti-PD-L1 or αPDL1_NG_). Based on the variable regions of atezolizumab, we designed an anti-PD-L1 antibody construct comprising N-glycosylation sites in its Fc region and expressed it in two different cell lines of the human expression platform GlycoExpress leading to anti-PD-L1 antibody variants with different glycosylation patterns: first, an anti-PD-L1 antibody with core fucosylated N-glycans (referred to as wild-type anti-PD-L1 or αPDL1_WT_) and second, an anti-PD-L1 antibody with minimized core fucosylation (referred to as glyco-engineered anti-PD-L1 or αPDL1_GE_).

To confirm the different glycosylation pattern of αPDL1_WT_ and αPDL1_GE_ we isolated the N-linked oligosaccharides from both variants and ran mass spectrometric glycan analyses. Both variants had comparable levels of N-glycans bearing galactose, sialic acid, or bisecting *N*-acetylglucosamine. However, an obvious difference was detected in the degree of fucosylation with 92% for αPDL1_WT_ and only 4% for αPDL1_GE_ (Table 1).

**Table 1:** N-glycan analysis of the anti-PD-L1 variants αPDL1_WT_ and αPDL1_GE_

Antibody	F (%)	B (%)	S > 0 (%)	G > 0 (%)
αPDL1_WT_	92	2	5	56
αPDL1_GE_	4	3	7	58

*The table summarizes the relative molar amounts of individual N-glycan structures to the following main glycan parameters: F = core fucosylated N-glycans; B = N-glycans bearing bisecting N-acetylglucosamine; S > 0 = sialylated N-glycans; G > 0 = galactosylated N-glycans*.

**Figure 1:**
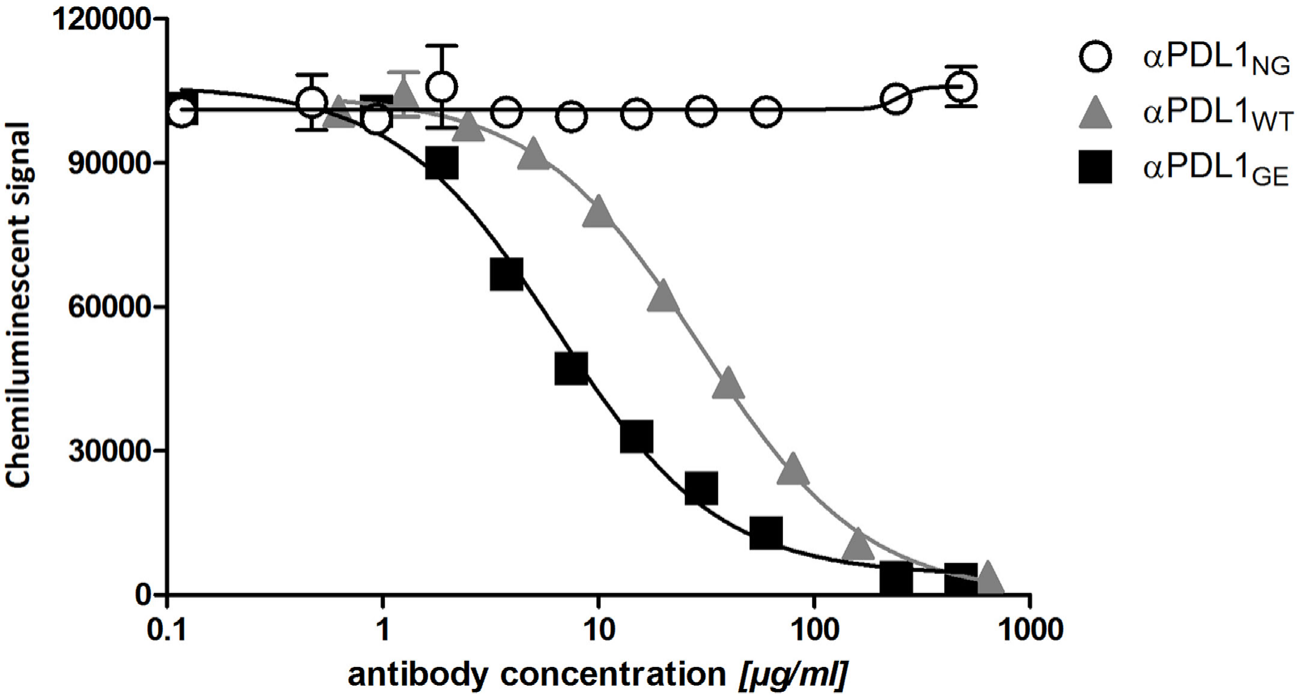
Glyco-engineered anti-human programmed death-ligand 1 (PD-L1) antibody shows an enhanced binding to FcγRIIIa. A competitive FcγRIIIa AlphaLISA was performed for the three anti-PD-L1 variants. Thereby the test antibody competes with antibody-conjugated acceptor beads for binding to FcγRIIIa-conjugated donor beads. The chemiluminescent signal as a result of close proximity of the donor and acceptor beads was plotted against increasing concentrations of αPDL1_NG_ (open circles), αPDL1_WT_ (gray triangles), and αPDL1_GE_ (black squares). Statistics: mean and SD were plotted in the graph. Data are representative of two independent experiments.

The removal of core fucose from N-glycans of the Fc region typically leads to increased antibody affinity to FcγRIIIa (21). To test for differences in the binding capacities of αPDL1_NG_, αPDL1_WT_, and αPDL1_GE_ to hFcγRIIIa, we used the bead-based AlphaScreen technology. The obtained data confirm that αPDL1_GE_ shows enhanced binding compared to αPDL1_WT_, while αPDL1_NG_ completely lacks FcγRIIIa binding (Figure 1). This is reflected by a reduced EC50 value for αPDL1_GE_ compared to αPDL1_WT_ and a relative potency of 3.98 demonstrating ~fourfold enhanced binding to FcγRIIIa (Table S1 in Supplementary Material). Thus, we had three anti-PD-L1 glycosylation variants with different affinities for hFcγRIIIa available for comparative functional *in vitro* tests.

### Antigen Binding of Glyco-Engineered Anti-PD-L1 Antibody Is Comparable to Its Normal and Non-Glycosylated Counterparts

**Figure 2:**
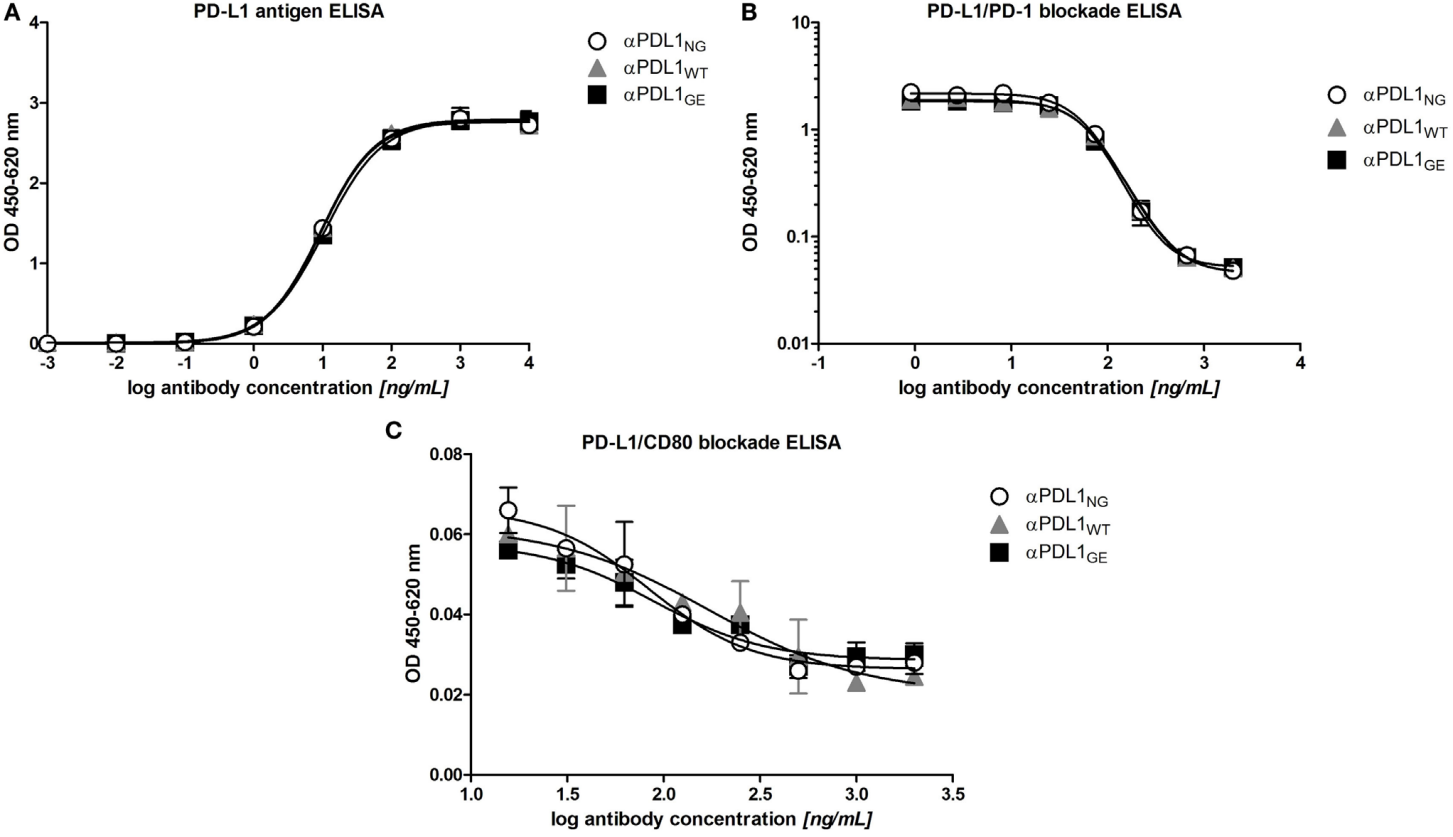
Glyco-engineered anti-human programmed death-ligand 1 (PD-L1) antibody and its normal and non-glycosylated counterparts show comparable antigen binding characteristics. The three anti-PD-L1 variants αPDL1_NG_ (open circles), αPDL1_WT_ (gray triangle), and αPDL1_GE_ (black squares) were tested for PD-L1 antigen binding and their capacity to block interaction with PD-L1 ligands in enzyme-linked immunosorbent assays (ELISA). **(A)** PD-L1 antigen binding ELISA. OD_450–620_ values were plotted against increasing concentrations of test antibody to assess binding to plate-bound human PD-L1. **(B)** Competitive ELISA measuring binding of soluble programmed death 1 to plate-bound PD-L1 in presence of test antibody. OD4_450–620_ values were plotted against increasing concentrations of test antibody. **(C)** Competitive ELISA measuring binding of soluble CD80 to plate-bound PD-L1 in presence of test antibody. OD_450–620_ values were plotted against increasing concentrations of test antibody. Statistics: mean and SD were plotted in all graphs. Data are representative of two independent experiments.

As prerequisite for subsequent comparison in functional assays we analyzed the antigen binding properties of αPDL1_NG_, αPDL1_WT_, and αPDL1_GE_ using ELISA and flow cytometry. Binding to plate-bound PD-L1 (Figure 2A) and blocking of the interaction with PD-1 and CD80 (Figures 2B,C) was comparable for all three variants. Furthermore, a cross-reactivity ELISA revealed an identical binding pattern to PD-L1 from other species (atezolizumab is cross-reactive with mouse, rat, and cynomolgus monkey PD-L1), whereas no binding to other human B7 family members (PD-L2, CD80, CD86, B7-H3, B7-H4, and B7-H5) was observed for any of the three variants (Figure S1 in Supplementary Material). An identical cell surface binding pattern of the three anti-PD-L1 variants as determined for the human cell lines DU-145 (PD-L1^+^) and Ramos (PD-L1^–^) by flow cytometry substantiated the ELISA results (Figure S2 in Supplementary Material). Collectively, our data confirm similar PD-L1 binding properties for the three different anti-PD-L1 variants which were expected as they share identical complementary determining regions.

### Glyco-Engineered Anti-PD-L1 Antibody Mediates Strong ADCC Against PD-L1^+^ Cancer Cells, but Not Against B Cells and Monocytes

**Figure 3:**
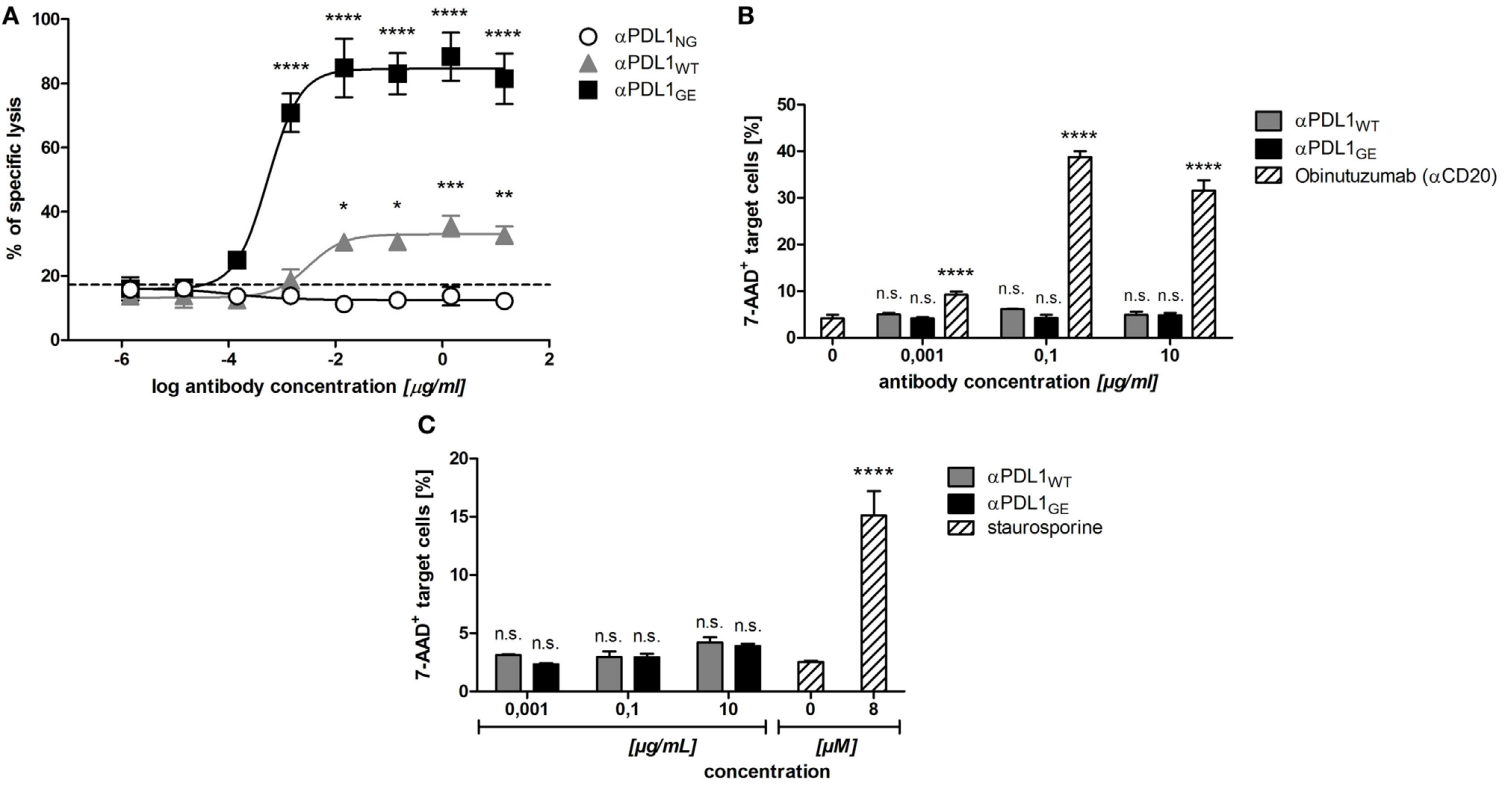
Glyco-engineered anti-human programmed death-ligand 1 (PD-L1) antibody induces strongest NK cell-mediated antibody dependent cellular cytotoxicity (ADCC) against PD-L1^+^ cancer cells, but none against B cells and monocytes. **(A)** The NK cell line KHYG-1-CD16aV as effector cells was incubated with europium-loaded PD-L1^+^ DU-145 cancer cells as target cells in an effector to target ratio of 10:1 in the presence of increasing concentrations of αPDL1_NG_ (white circles), αPDL1_WT_ (gray triangles), or αPDL1_GE_ (black squares) for 5 h to determine the lysis of target cells in an *in vitro* cytotoxicity assay. The percentage of specific target cell lysis is plotted against the antibody concentration used. The dashed line indicates % of lysis in the medium control. **(B,C)** The NK cell line KHYG-1-CD16aV as effector cells were incubated with calcein-labeled primary B cells **(B)** or monocytes **(C)** as target cells in an effector to target ratio of 10:1 in the presence of increasing concentrations of αPDL1_WT_ (gray bars) or αPDL1_GE_ (black bars) for 4 h to determine the killing of target cells in a flow cytometry based *in vitro* cytotoxicity assay. The relative frequency of dead 7-AAD^+^ of calcein^+^ target cells was plotted against the antibody concentration used. As a positive control (striped bars) either obinutuzumab (αCD20) was used to induce B cell lysis or staurosporine was used to induce monocyte lysis. Statistics: mean and SD were plotted in all graphs. Data are representative of two independent experiments. Significance was tested against the medium control (*p < 0.05; **p < 0.01; ***p < 0.001; and ****p < 0.0001. Abbreviation: ns., not significant).

Increased affinity to FcγRIIIa due to reduced core fucosylation typically results in enhanced ADCC (21). In order to test if the observed differences in FcγRIIIa binding translate into increased ADCC activity we compared the three anti-PD-L1 variants in an ADCC assay with PD-L1^+^ DU-145 as targets and a FcγRIIIa-expressing NK cell line as effectors. As expected due to eliminated Fc-effector functions, no ADCC activity was observed in presence of αPDL1_NG_. In contrast, αPDL_WT_ mediated target cell lysis and the effect was drastically enhanced by αPDL1_GE_ (Figure 3A).

Since αPDL_WT_ and αPDL1_GE_ effectively mediated ADCC against cancer cells, we investigated their ADCC effect on PBMC subsets. We identified B cells and monocytes as PBMC subpopulations partially expressing PD-L1 (Figure S3 in Supplementary Material) and thus tested their sensitivity toward anti-PD-L1-mediated ADCC. The positive control obinutuzumab (anti-CD20) effectively induced lysis of primary B cells, whereas neither αPDL1_WT_ nor αPDL_GE_ showed any ADCC effect (Figure 3B). Similarly, no obvious lysis of monocytes was induced by both glycosylated anti-PD-L1 variants, whereas the positive control staurosporine was effective (Figure 3C). In summary, the glyco-engineered anti-PD-L1 variant induced increased ADCC against PD-L1^+^ cancer cells compared to the normal glycosylated counterpart, but no unwanted ADCC was observed against B cells and monocytes.

### Glyco-Engineered Anti-Human PD-L1 Antibody Induces Enhanced CD8 T Cell Activation

**Figure 4:**
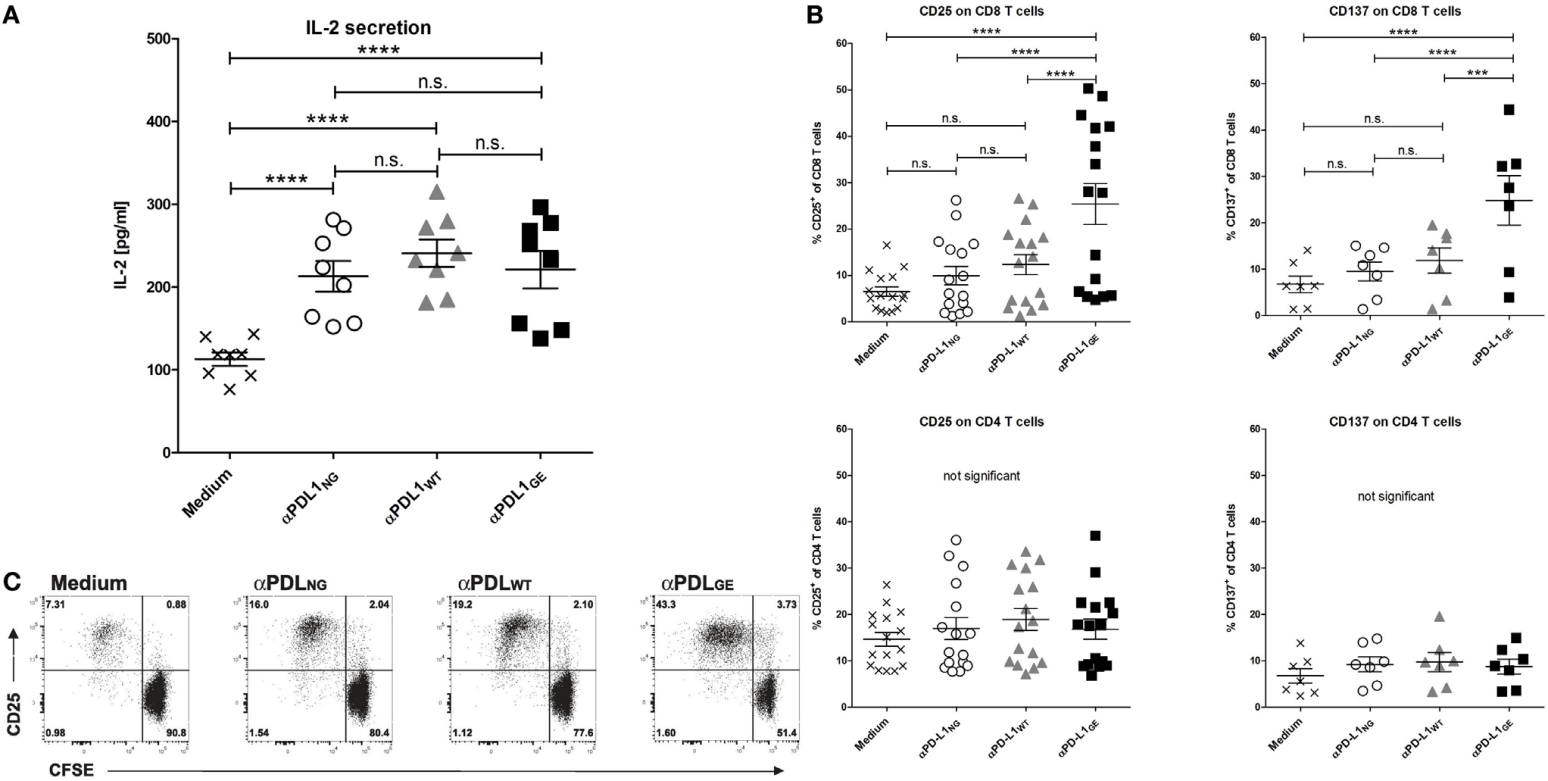
Glyco-engineered anti-human programmed death-ligand 1 (PD-L1) antibody induces strong CD8 T cell activation in a mixed leukocyte reaction. The three anti-PD-L1 variants αPDL1_NG_ (open circles), αPDL1_WT_ (gray triangles), and αPDL1_GE_ (black squares) were tested for their effect on T cell activation in a mixed leukocyte reaction (MLR). The medium control (black crosses) served as a negative control. T cells as responder cells were isolated from a single healthy donor (donor A). Monocyte-derived dendritic cells as stimulator cells were generated from different healthy donors. **(A)** IL-2 enzyme-linked immunosorbent assays on day 2 of MLR. The determined concentrations of IL-2 in the culture supernatants were plotted. **(B)** The activation status of CD8 and CD4 T cells in the MLR was determined on day 5 by flow cytometric analysis. The relative frequencies of CD25^+^ and CD137^+^ in CD8 and CD4 T cells were plotted. **(C)** The proliferation of CFSE-labeled CD8 T cells in the MLR was determined on day 5 by CFSE dilution measured by flow cytometric analysis. Representative plots of CD25 expression and CFSE signal intensity of CD8 T cells are shown. Statistics for **(A,B)**: besides individual data points (*n* = 8 for IL-2 secretion, *n* = 16 for CD25 expression, and *n* = 7 for CD137), mean and SEM were plotted in all graphs (*p < 0.05; **p < 0.01; ***p < 0.001; and ****p < 0.0001. Abbreviation: ns, not significant).

The anti-tumor effect of PD-1/PD-L1 inhibitors is primarily based on restoration of T cell activity (1). An accepted method to investigate the ability of PD-1/PD-L1 checkpoint inhibitors to activate T cells *in vitro* is the allogeneic MLR, where moDCs and T cells from different donors are co-incubated to mimic immunosuppressive effects by the interaction of PD-L1 and PD-1 (14, 29). Figure S4 in Supplementary Material shows the typical phenotype of moDCs used for the MLRs. As responders, T cells were isolated from PBMCs with high purity ranging from 86 to 95% (determined as CD3^+^ CD56^−^ cells). When analyzing the MLR supernatants for IL-2 after 2 days, it was found that all three anti-PD-L1 variants restored the secretion of IL-2 by T cells to a similar extent (Figure 4A). On day 5 of the MLR, CD8 T cells were analyzed for surface expression of CD25 (IL-2Rα) and CD137 (4-1BB, Tnfrsf9) by flow cytometry to determine their activation status. While the addition of αPDL1_NG_ and αPDL1_WT_ to the MLR resulted in minimally elevated CD8 T cell activation compared to the medium control, incubation with αPDL1_GE_ induced significantly enhanced CD8 T cell activation (Figure 4B). The increased activation was accompanied by enhanced proliferation of CD8 T cells (Figure 4C). Interestingly, none of the anti-PD-L1 variants was able to induce an obvious activation of CD4 T cells in terms of upregulation of CD25 and CD137 (Figure 4B) and proliferation (data not shown). Furthermore, we examined the expression of the maturation/activation molecules CD80 (B7-1), CD86 (B7-H2), and HLA-DR on moDCs on day 5 of the MLR (Figure S5 in Supplementary Material). In particular, CD86 and HLA-DR were found to be upregulated in presence of αPDL1_GE_ suggesting a more mature status of the moDCs.

Since we observed different results between the anti-PD-L1 variants with respect to T cell activation marker expression on day 5 but not for IL-2 secretion on day 2, we compared the expression of CD25 on CD8 T cells on day 2 and day 5 (Figure S6-1 in Supplementary Material). Although overall CD8 T cell activation was obviously higher on day 5 compared to day 2, the advantage of αPD-L1_GE_ was detectable on both days. In addition, Figure S6-2 and Table S2 in Supplementary Material illustrates that the advantage in CD8 T cell activation induced by αPDL1_GE_ is present over a range of different antibody concentrations and observed using PBMCs or isolated T cells from further donors.

**Figure 5:**
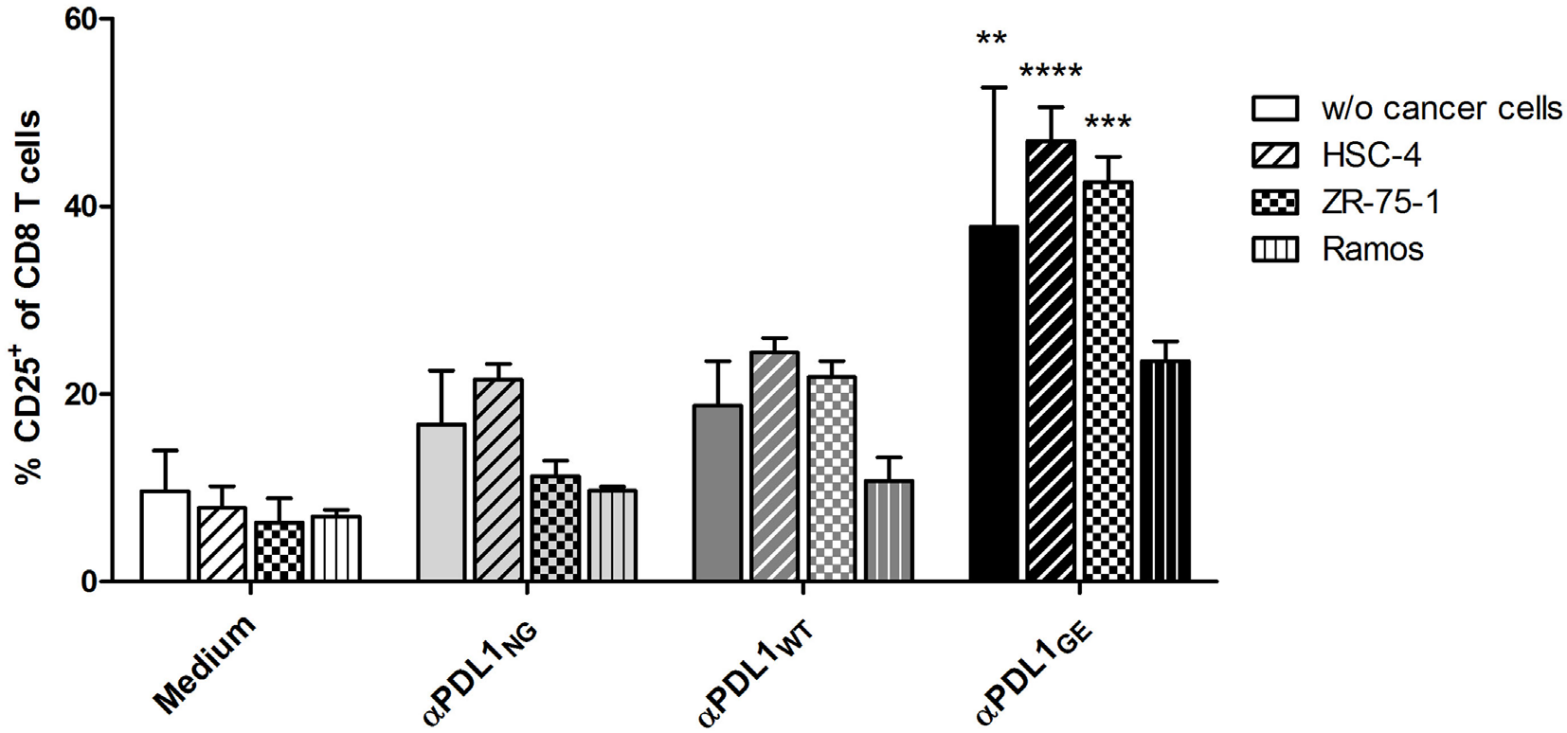
Glyco-engineered anti-human programmed death-ligand 1 (PD-L1) antibody induces increased CD8 T cell activation in presence of cancer cells. The three anti-PD-L1 variants αPDL1_NG_ (light gray bar), αPDL1_WT_ (dark gray bar), and αPDL1_GE_ (black bar) were tested for their effect on T cell activation (donor A) in allogeneic mixed leukocyte reaction (MLRs) in absence or presence of the cancer cell lines HSC-4 (horizontal stripes), ZR-75-1 (plaid), and Ramos (vertical stripes). MLR without addition of test antibody (medium; white bar) served as negative control. The relative frequencies of CD25^+^ in CD8 T cells were plotted. Statistics: mean and SD were plotted in all graphs. Data are representative of two independent experiments. Significance was tested against the medium control without presence of cancer wells (*p < 0.05; **p < 0.01; ***p < 0.001; and ****p < 0.0001).

To confirm these differences in T cell activation in a more physiological setting, MLRs were performed in the presence of different cancer cell lines. The head and neck cancer cell line HSC-4, the breast cancer line ZR-75-1, and Ramos originally derived from B-cell lymphoma, respectively, were added on day 0 to the MLR (T cells:moDC:cancer cells = 100:10:1). Measuring CD25 expression revealed that the presence of HSC-4 and ZR-75-1 had no obvious effect on the CD8 T cell activation, whereas Ramos cells appear to have some suppressive impact (Figure 5).

In summary, while a normal glycosylated variant of anti-PD-L1 is able to induce T cell activation to a similar level compared to the non-glycosylated antibody, the glyco-engineered variant results in robustly enhanced CD8 T cell activation.

### Increased T Cell Activation by Glyco-Engineered Anti-PD-L1 Antibody Translates Into Higher Cytotoxicity Against Cancer Cells

**Figure 6:**
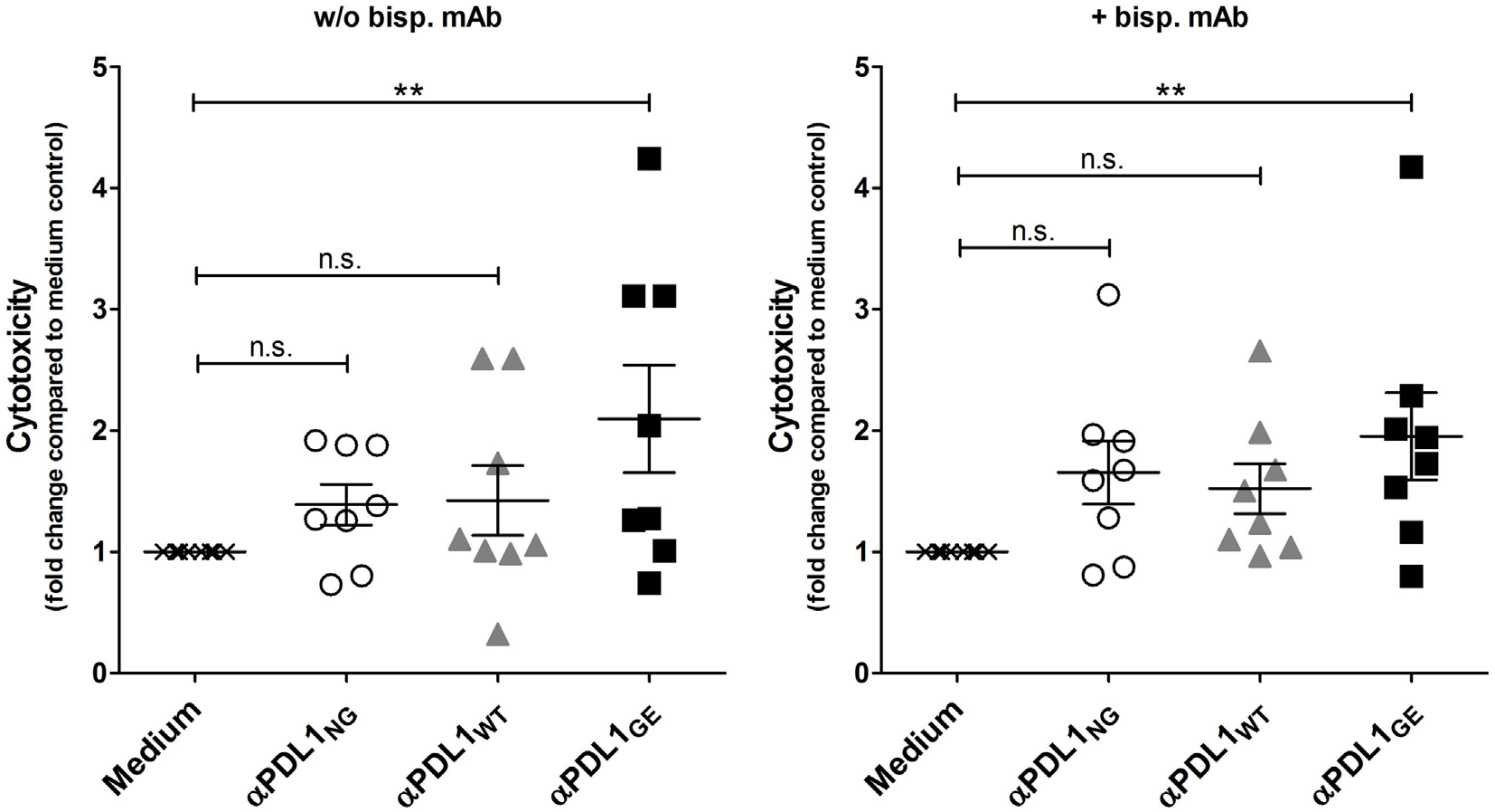
αPDL1_GE_-dependent enhancement of CD8^+^ T cell activation results in increased cytotoxicity against cancer cells. T cells of two different donors (donor B and donor C) pre-activated in a mixed leukocyte reaction (MLR) for 5 days in the presence of αPDL1_NG_ (open circles), αPDL1_WT_ (gray triangles), and αPDL1_GE_ (black squares) were harvested and incubated with europium-loaded ZR-75-1 cells for 5 h to determine the lysis of target cells in an *in vitro* cytotoxicity assay. T cells isolated from MLR without addition of test antibody (medium; black crosses) served as negative control. The cytotoxicity assay was performed in absence and presence of a bispecific antibody binding to a tumor antigen on ZR-75-1 and to CD3 on T cells. The fold change in cytotoxicity compared to the medium control is plotted. Statistics: besides individual data points (*n* = 8), mean and SEM were plotted. Significance was tested against the medium control (*p < 0.05; **p < 0.01; ***p < 0.001; and ****p < 0.0001).

We also addressed the question, if the enhanced T cell activation induced by the glyco-engineered anti-PD-L1 antibody has an influence on T cell effector functions, in particular cytotoxicity against cancer cells. For that purpose, T cells which were activated in a MLR in absence or presence of αPDL1_NG_, αPDL1_WT_, and αPDL1_GE_ were harvested and analyzed for their cytotoxic capacity using a europium release assay. Therefore, we selected ZR-75-1 as target cells because they were not sensitive for anti-PD-L1-mediated ADCC (Figure S7 in Supplementary Material). Since we assumed that T cells isolated from the MLR were not particularly reactive to ZR-75-1 due to lack of specificity, we additionally tested their cytotoxicity in presence of a bispecific antibody binding to a tumor antigen on ZR-75-1 and to CD3 on T cells ensuring close proximity between target and effector cell. In both conditions, in absence and presence of the bispecific antibody, the cytotoxicity of pre-activated T cells was increased compared to the medium control, and the strongest pre-activation due to treatment with αPDL1_GE_ resulted in the highest cytotoxicity (Figure 6; Figure S8 in Supplementary Material).

## Discussion

Programmed death-ligand 1 blockade using therapeutic anti-PD-L1 antibodies is a promising approach in the therapy of a variety of cancers as it interferes with a co-inhibitory checkpoint pathway on TILs and thereby enhances anti-tumor immunity (1). In line with findings for other immunomodulatory antibodies, e.g., anti-CTLA-4 (30), recent preclinical data obtained in a syngeneic mouse tumor model highlight the contribution of the Fc domain for the functionality of anti-PD-L1 antibodies and suggests that the anti-tumor response of anti-PD-L1 antibodies is at least partially dependent on engagement of activating FcγRs (13). Thus, we generated an anti-PD-L1 of hIgG1 isotype based on the variable regions of non-glycosylated atezolizumab bearing “normal” core fucosylated N-glycans in its Fc region and a further glyco-engineered variant with fucose-reduced N-glycans. When comparing the non-glycosylated, the normal glycosylated and the glyco-engineered anti-PD-L1 variants we observed identical antigen binding, but enhanced FcγRIIIa binding for αPDL1_GE_ > αPDL1_WT_ > αPDL1_NG_. Accordingly, NK cell-mediated ADCC against a PD-L1^+^ cancer cell line was enhanced for αPDL1_GE_ > αPDL1_WT_ > αPDL1_NG_. In contrast, no ADCC against PD-L1 expressing B cells and monocytes could be detected. Surprisingly, testing of these anti-PD-L1 variants in a MLR revealed increased CD8 T cell activation potential for αPDL1_GE_ > αPDL1_WT_ ≥ αPDL1_NG_ determined by surface expression of CD25 and CD137, proliferation as well as cytotoxicity against cancer cells.

Tumor cell killing *via* ADCC is an important mechanism of several therapeutic antibodies including rituximab and cetuximab as demonstrated in human *in vitro* systems (31, 32) and as implicated by several clinical studies (33, 34). Avelumab which is to our knowledge a normal glycosylated anti-PD-L1 antibody has been shown to mediate ADCC in *in vitro* studies (24). Similarly, we generated the normal glycosylated anti-PD-L1 antibody to allow for mediation of ADCC *via* a functional Fc part. For our glyco-engineered anti-PD-L1 antibody we found an enhanced NK cell-mediated ADCC against PD-L1^+^ cancer cells compared to the normal glycosylated variant, which is in line with the well-established superior ADCC capacity of several glyco-engineered antibodies (e.g., anti-CD20, anti-EGFR) (32, 35). However, an enhanced capability for ADCC involves the risk of unwanted cytotoxic effects on PD-L1-expressing PBMC. Avelumab is described to mediate no obvious lysis of PBMCs as shown by *in vitro* cytotoxicity assays and by the fact that patients treated with avelumab had no significant changes in their peripheral immune cell profile (24, 36). In addition, avelumab has a manageable safety profile in the clinic (18) as it is also reported for the anti-PD-L1 antibodies which are unable to mediate ADCC (17, 19). In line with literature data (2), we identified B cells and monocytes as PBMC subpopulations partially expressing PD-L1. Subsequent testing revealed that neither the normal glycosylated nor the glyco-engineered anti-PD-L1 antibody mediated any measurable lysis of these cells consistent with the results shown for avelumab (24, 36). Since the antigen density on the cancer cell surface impacts the anti-PD-L1 antibody mediated ADCC (24), we suppose that the expression level of PD-L1 on the tested PBMC subpopulations is too low for effective lysis of these cells. However, we cannot generally exclude that PD-L1-expressing immune cells will not be targeted by anti-PD-L1 antibodies and for that reason a potential ADCC against immune cells needs to be thoroughly investigated by further tests, e.g., by using pre-activated PBMC as targets. Since myeloid-derived suppressor cells (MDSCs) in the tumor microenvironment have increased expression of PD-L1 (37), an antibody dependent depletion of these immune cells might also be beneficial as it is already described by Dahan and colleagues in their syngeneic mouse model (13).

To our knowledge this is the first demonstration that glyco-engineering of an anti-PD-L1 antibody results in increased CD8 T cell activation conferring to an enhanced CD8 T cell cytotoxicity against tumor cells. It is known that removal of fucose from N-glycans of the Fc portion of hIgG primarily results in enhanced binding to FcγRIIIa (21). In combination with our finding that the tested anti-PD-L1 variants differed with respect to FcγRIIIa but not antigen binding, we assume that binding of the Fc part to FcγRIIIa is causative for the observed differences in the strength of CD8 T cell activation in the MLR. Thus our study provides a further indication in addition to the results of Dahan and colleagues (13) that FcγR engagement augments the anti-tumor activity of anti-PD-L1 antibodies.

It has been shown that PD-1/PD-L1 blockade results in IL-2 secretion of T cells (29). Interestingly, (using an allogeneic MLR) addition of all three anti-PD-L1 variants heightened the IL-2 release in a similar range. In contrast, CD8 T cell activation (according to surface expression of CD25 and CD137) was most pronounced in the presence of the glyco-engineered anti-PD-L1 antibody. IL-2 is typically produced by T helper cells (also referred to as CD4 T cells), although it can also be produced by cytotoxic T cells (also referred to as CD8 T cells) but to a lesser extent. Since we did not observe obvious differences in IL-2 secretion and in CD4 T cell activation regarding the expression of CD25 and CD137 between the different glycosylation variants, we assume that IL-2 detected in the MLR was primarily secreted by CD4 T cells and that glyco-engineering has a stronger effect on CD8 T cell activation than on CD4 T cells.

Studies in syngeneic mouse models revealed that engagement of activating FcγRs by anti-PD-L1 antibodies augments their anti-tumor activity and that this effect was partially mediated by depletion of PD-L1-expressing MDSC (13). Similar observations were reported for anti-CTLA-4, anti-GITR, and anti-OX40 antibodies in murine systems demonstrating that binding to activating FcγR results in depletion of intra-tumoral Tregs and contributes to anti-tumor activity (30, 38–40). Simpson and colleagues identified FcγRIV-expressing macrophages as potential effector cells mediating ADCC against CTLA-4-expressing intra-tumoral Tregs (39). Human FcγRIIIa is the ortholog of murine FcγRIV (22) suggesting that FcγRIIIa-bearing immune cells might represent corresponding effector cells in the human system. Indeed, melanoma patients responding to anti-CTLA-4 therapy (ipilimumab, hIgG1) displayed increased levels of circulating nonclassical FcγRIIIa-expressing monocytes able to lyse Tregs *ex vivo*, and additionally showed a decrease in intra-tumoral Tregs after treatment with concurrent presence of FcγRIIIa-expressing M1-like macrophages in the tumor (41). Monocytes might also play a role in our MLR experiments as it was set up with purified *in vitro* differentiated monocyte-derived DC (moDC) as stimulators and potentially some remaining undifferentiated FcγRIIIa^+^ monocytes might eliminate PD-L1^+^ suppressor cells and thereby enhance the response. Furthermore, monocytes might particularly act as cytotoxic cells lysing PD-L1^+^ suppressor cells in the MLR assays where total PBMCs were used as responder cells.

Since the glyco-engineered variant of anti-PD-L1 is generally capable of eliciting ADCC, also other FcγRIIIa^+^ immune cells besides monocytes might mediate ADCC-dependent depletion of PD-L1^+^ cells in the MLR setting and thereby contribute to the enhanced CD8 T cell activation. Beside NK cells, NKT cells and γδ-T cells are described to express FcγRIIIa and to mediate ADCC (42, 43). This could have a significant impact on the results in the MLR assays where whole PBMCs have been used as responders (NK cells, NKT cells, and γδ-T cells are present), but also when using isolated T cells (NKT cells and γδ-T cells still present). A systematic comparison of using isolated αβ-T cells ± defined FcγRIIIa^+^ immune cell subpopulations versus total PBMCs of the same donor as responders would address this issue, but was beyond the scope of this work.

Another possible explanation is that an enhanced occupancy of FcγRs by the glyco-engineered anti-PD-L1 variant results in a better maturation of moDCs followed by enhanced activation of CD8 T cells. Supporting this explanation, it was shown that anti-EGFR antibodies can directly bind to FcγR on monocytes leading to upregulation of co-stimulatory molecules (44), but also that anti-EGFR antibodies can initiate T cell responses *via* Fc-dependent activation of NK cells inducing DC maturation (44, 45). Further, γδ-T cells have been shown to secrete IFN-γ after FcγRIIIa engagement of antibodies (42), and thereby induce DC maturation resulting in enhanced CD8 T cell activation (46). In line with these studies, we observed an upregulated expression of the maturation markers CD86 and HLA-DR on the moDCs in presence of the glyco-engineered anti-PD-L1 antibody.

Two of the approved anti-PD-L1 antibodies were specifically designed to eliminate ADCC activity (14, 23), only one has Fc-mediated functionality (24) thereby combining blockade of the PD-1/PD-L1 axis and a limited ADCC activity in one molecule. Both modes of action might act synergistically. Blocking the PD-1/PD-L1 axis can enhance general anti-tumor activity of NK cells expressing PD-1 including direct cytotoxicity and ADCC against tumor cells (47, 48). The efficacy of antibodies targeting the PD-1/PD-L1 axis seems to be dependent on PD-L1 expression on the tumor (19). NK cells, but also γδ-T cells, activated through their FcγRs secrete IFN-γ (42) which in turn can upregulate PD-L1 expression on cancer cells (9). Since our data show that a glyco-engineered anti-PD-L1 antibody even combines a full blockade of the PD-1/PD-L1 axis with an increased CD8 T cell activation and an enhanced ADCC activity, the synergism between these modes of action might be even stronger. Furthermore, glyco-engineering of antibodies is expected to lead to a broader patient coverage, since enhanced FcγRIIIa binding is also observed for the low-affinity variant FcγRIIIa 158F of the receptor which is homozygous in ~42% (F/F) and heterozygous in ~50% (F/V) of healthy Caucasians (35, 49).

In summary, the present study demonstrates that glyco-optimization due to defucosylation has the potential to improve the therapeutic benefit of anti-PD-L1 antibodies. A glyco-engineered anti-PD-L1 antibody could be clinically developed as a monotherapeutic drug or as a combination therapy. Several clinical trials are underway for anti-PD-L1 antibodies in combination with other drugs, for example, with antibodies targeting stimulatory checkpoint molecules, with VEGF inhibitors or with different tyrosine kinase inhibitors (50). Our novel concept is the combination of a glyco-engineered anti-PD-L1 antibody with a second specificity directed against a tumor-specific antigen composed of a bispecific antibody construct, which would have the advantage that release of the PD-1/PD-L1 axis would be focused on the tumor site thereby potentially reducing side effects and increasing efficacy. The development and characterization of such a bispecific anti-PD-L1 antibody is currently ongoing.

## Author Contributions

Conception and design of project: CG, TL, JR, AD, and SG. Development of methodology: CG, JR, and TL. Acquisition of data: CG and TL. Analysis and interpretation of data: CG, TL, and UH. Drafting and revising the article: CG, TL, UH, PS, JR, AD, and SG.

## Conflict of Interest Statement

CG, TL, UH, PS, AD, JR, and SG are/were employed by and AD and SG are shareholders of Glycotope GmbH.
