# Supplementary material for: Glyco-Engineered Anti-Human Programmed Death-Ligand 1 Antibody Mediates Stronger CD8 T Cell Activation Than Its Normal Glycosylated and Non-Glycosylated Counterparts
Source: Front Immunol. 2018 Jul 16;9:1614. doi: 10.3389/fimmu.2018.01614 (PMC6054930; doi:10.3389/fimmu.2018.01614)
Supplement: Supplementary file 1 [file data_sheet_1.docx]

Supplementary Material

Glyco-engineered anti-human PD-L1 antibody mediates stronger CD8 T cell activation than its normal glycosylated and non-glycosylated counterparts

Christoph Goletz*, Timo Lischke, Ulf Harnack, Phillip Schiele, Antje Danielczyk, Johanna Rühmann, and Steffen Goletz

*** Correspondence:** Christoph Goletz: Christoph.Goletz@glycotope.com

# Supplementary Tables

**Supplementary Table 1:** Summary of FcγRIIIa binding of αPDL1_WT_ and αPDL1_GE_

| **antibody** | **EC50 *[µg/ml]*** | **relative potency** |
| --- | --- | --- |
| αPDL1_WT_ | 27.12 | - |
| αPDL1_GE_ | 6.81 | 3.98 |

|  | **% CD25+ of ...** | **Medium** | | | **αPDL1_NG_** | | | | **αPDL1_WT_** | | | | **αPDL1_GE_** | | | |
| --- | --- | --- | --- | --- | --- | --- | --- | --- | --- | --- | --- | --- | --- | --- | --- | --- |
|  |  | **Mean** | SEM | N | **Mean** | SEM | N |  | **Mean** | SEM | N |  | **Mean** | SEM | N |  |
| **total PBMC from donor A** | **CD8 T cells** | **7,7** | 1,5 | 2 | **11,7** | 5,4 | 2 | n.s. | **11,2** | 0,3 | 2 | n.s. | **53,4** | 1,4 | 2 | ** |
|  | **CD4 T cells** | **19,2** | 2,6 | 2 | **24,1** | 8,4 | 2 | n.s. | **20,2** | 0,5 | 2 | n.s. | **38,0** | 4,0 | 2 | n.s. |
| **isolated T cells from donor B** | **CD8 T cells** | **14,8** | 1,2 | 2 | **20,0** | 2,1 | 2 | n.s. | **26,2** | 0,3 | 2 | * | **41,5** | 0,6 | 2 | *** |
|  | **CD4 T cells** | **35,4** | 4,5 | 2 | **43,2** | 1,9 | 2 | n.s. | **47,5** | 0,4 | 2 | n.s. | **48,2** | 2,7 | 2 | n.s. |
| **isolated T cells from donor C** | **CD8 T cells** | **20,0** | 2,3 | 2 | **34,8** | 1,0 | 2 | n.s. | **42,8** | 2,1 | 2 | * | **52,1** | 4,5 | 2 | ** |
|  | **CD4 T cells** | **20,4** | 3,7 | 2 | **21,3** | 1,2 | 2 | n.s. | **27,9** | 4,7 | 2 | n.s. | **30,4** | 1,3 | 2 | n.s. |
| **total PBMC from donor D** | **CD8 T cells** | **6,1** | 0,7 | 2 | **10,9** | 3,0 | 2 | n.s. | **6,9** | 0,0 | 2 | n.s. | **43,7** | 1,4 | 2 | *** |
|  | **CD4 T cells** | **20,0** | 3,0 | 2 | **23,2** | 3,5 | 2 | n.s. | **16,7** | 0,9 | 2 | n.s. | **22,8** | 2,2 | 2 | n.s. |
| **total PBMC from donor E** | **CD8 T cells** | **0,9** | 0,5 | 2 | **2,6** | 0,6 | 2 | n.s. | **6,4** | 0,6 | 2 | n.s. | **7,3** | 1,6 | 2 | * |
|  | **CD4 T cells** | **5,1** | 1,0 | 2 | **8,9** | 0,5 | 2 | n.s. | **13,1** | 2,4 | 2 | n.s. | **9,7** | 0,1 | 2 | n.s. |
| **total PBMC from donor F** | **CD8 T cells** | **0,6** | 0,4 | 2 | **0,9** | 0,1 | 2 | n.s. | **3,3** | 0,6 | 2 | n.s. | **6,5** | 0,5 | 2 | ** |
|  | **CD4 T cells** | **7,9** | 1,4 | 2 | **8,3** | 0,0 | 2 | n.s. | **13,8** | 2,0 | 2 | n.s. | **13,7** | 0,5 | 2 | n.s. |
| **isolated T cells from donor G** | **CD8 T cells** | **13,4** | 0,5 | 2 | **25,2** | 3,7 | 2 | n.s. | **24,8** | 3,9 | 2 | n.s. | **33,6** | 0,8 | 2 | * |
|  | **CD4 T cells** | **22,6** | 0,5 | 2 | **40,5** | 3,3 | 2 | n.s. | **42,1** | 4,1 | 2 | * | **46,9** | 1,4 | 2 | * |
| **isolated T cells from donor H** | **CD8 T cells** | **23,0** | 4,0 | 2 | **34,1** | 1,5 | 2 | n.s. | **34,5** | 3,1 | 2 | n.s. | **43,1** | 0,4 | 2 | * |
|  | **CD4 T cells** | **44,0** | 2,5 | 2 | **52,5** | 0,3 | 2 | n.s. | **56,6** | 3,0 | 2 | n.s. | **55,4** | 0,1 | 2 | n.s. |

**Supplementary Table 2**: Summary of CD8 and CD4 T cell activation in MLR assays using different donors. The table shows the Mean, SEM and number of replicates of relative frequencies of CD25^+^ in CD8 T cells and CD4 T cells measured in MLR assays using isolated T cells of total PBMCs from different donors as responder cells. Significance was tested against the medium control. P values were determined by using a One-Way ANOVA and a Bonferroni post test (*p < 0.05; **p < 0.01; ***p < 0.001; ****p < 0.0001; ns = not significant).

# Supplementary Figures

**
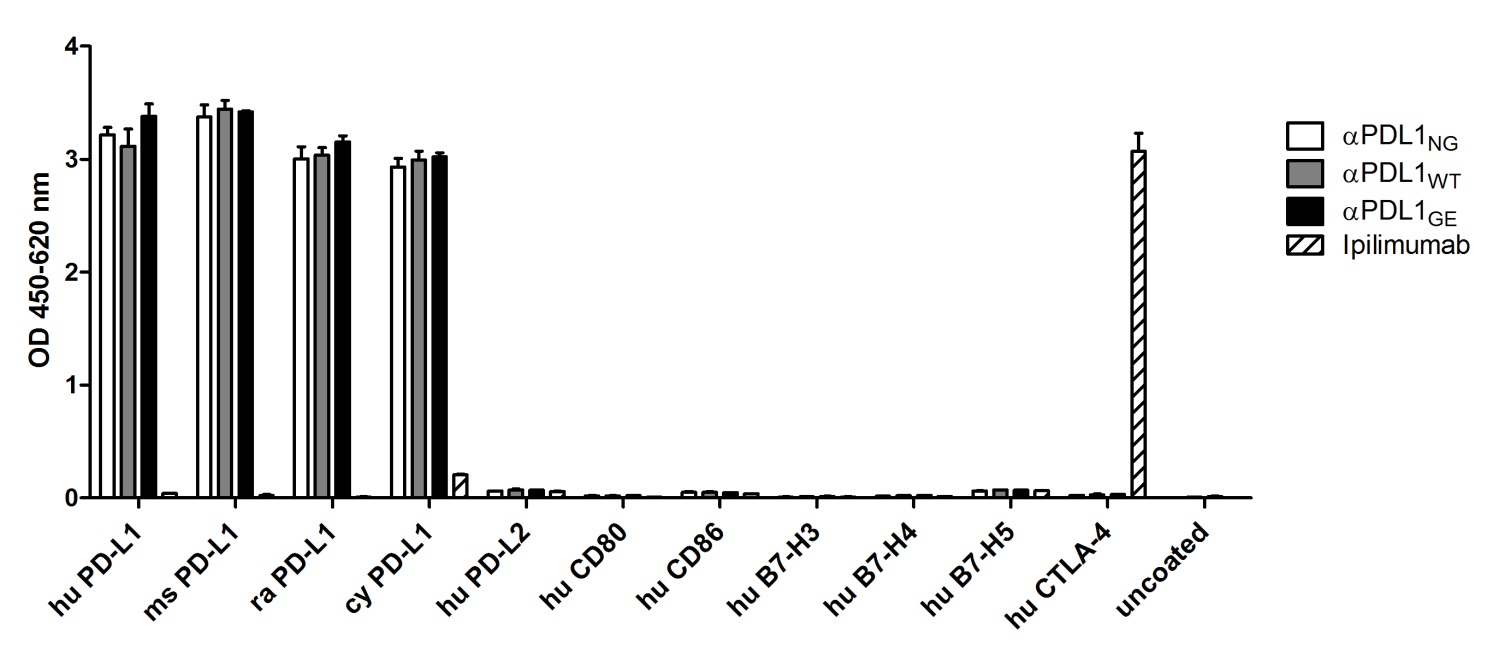
**

**Supplementary Figure 1:** Cross-reactivity ELISA for binding to PD-L1 from other species and for other B7 family members. OD_450-620_ values were plotted for αPDL1_NG_ (white), αPDL1_WT_ (gray), αPDL1_GE_ (black), and ipilimumab (anti-human CTLA-4, shaded) to show antibody binding of plate-bound PD-L1 from human, mouse, rat and cynomolgus monkey, as well as binding to plate-bound human PD-L2, CD80, CD86, B7-H3, B7-H4, B7-H5, and CTLA-4. Test antibodies were incubated with saturating concentrations and detected with a secondary goat anti-hu IgG Fc-POD antibody. Statistics: mean and SD of duplicates were plotted. Data are representative of two independent experiments.


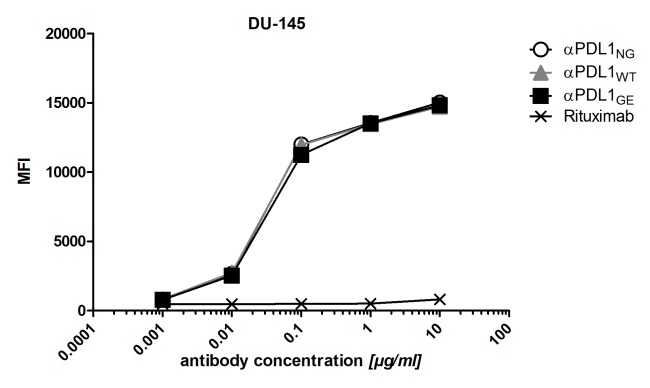

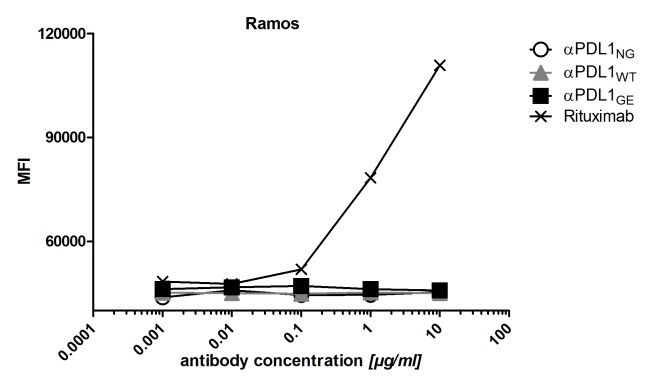


**Supplementary Figure 2:** Binding of anti-human PD-L1 variants to PD-L1^+^ DU-145 and PD-L1^–^ Ramos cells determined in flow cytometry. The median fluorescence intensity is plotted against increasing concentrations of αPDL1_NG_ (open circles), αPDL1_WT_ (gray triangles), αPDL1_GE_ (black squares), and rituximab (anti-human CD20; black crosses). Rituximab was used as a positive control for binding to CD20^+^ Ramos cells. Test antibodies were detected using a secondary F(ab)_2_ fragment goat anti-human IgG+IgM (H+L) PE-conjugated antibody. Statistics: mean and SD of duplicates were plotted in all graphs. Data are representative of two independent experiments.

**
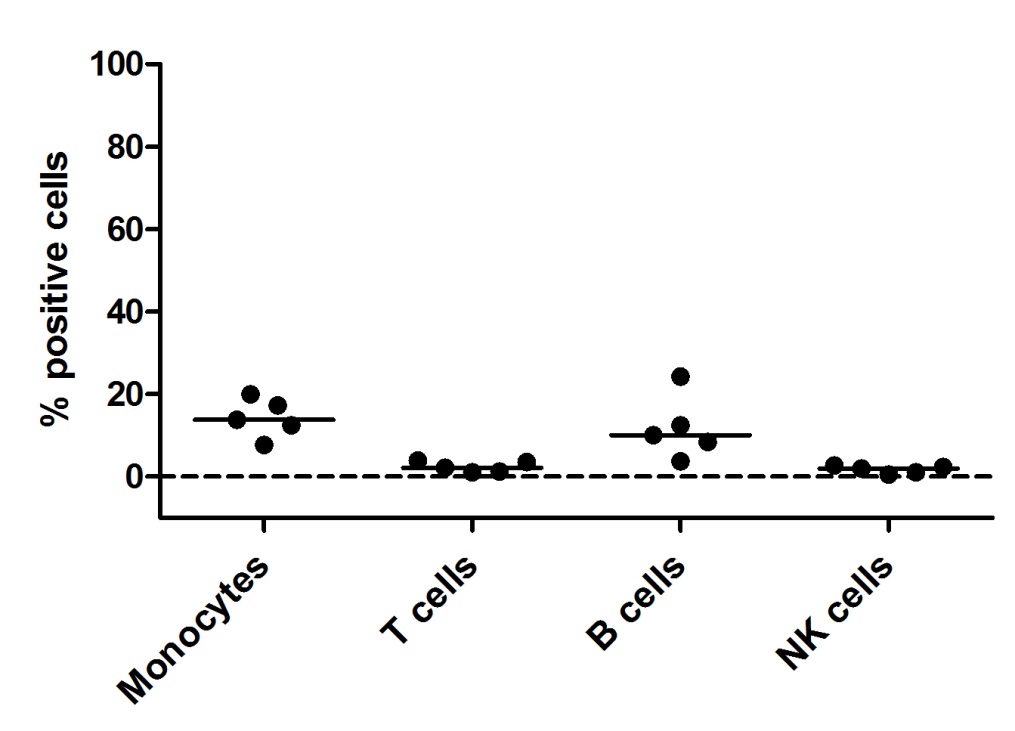
**

**Supplementary Figure 3**: Expression of PD-L1 on PBMC subsets. PD-L1 expression on different immune cell populations of PBMCs from five different healthy donors was analyzed by a flow cytometry using a biotinylated anti-PD-L1 antibody. PBMC were blocked with human IgG prior to staining. Binding of anti-PD-L1 antibody was detected with Streptavidin-PE. Co-staining with fluorescence-labelled anti-CD19, -CD3, -CD14 and -CD56 allowed for gating on monocytes, T cells, B cells and NK cells, respectively. Statistics: besides individual data points, the median was plotted.


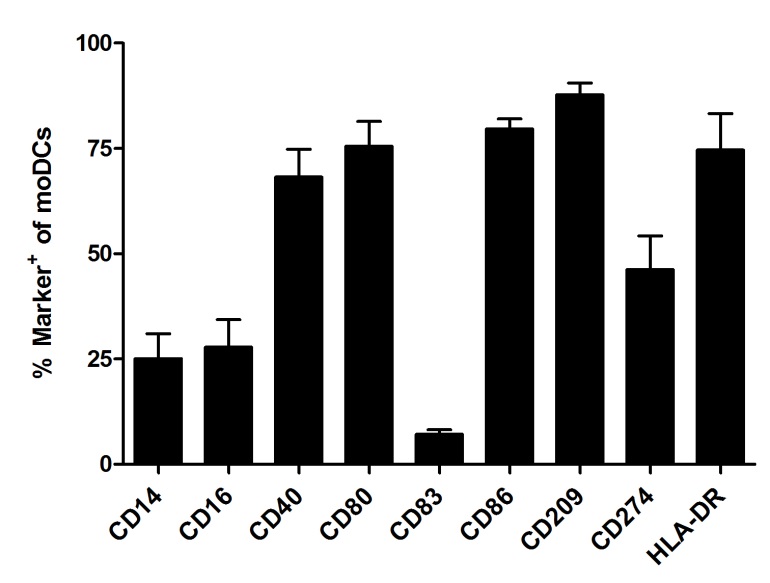


**Supplementary Figure 4**: Phenotype of monocyte-derived dendritic cells. The monocyte-derived dendritic cells (moDC) used for the MLR were phenotyped by flow cytometry according to expression of various differentiation molecules. The relative frequencies of CD14^+^, CD16^+^, CD40^+^, CD80^+^, CD83^+^, CD86^+^, CD209^+^, CD274^+^, and HLA-DR^+^ cells were plotted. Statistics: mean and SEM of ten individual experiments were plotted.

**
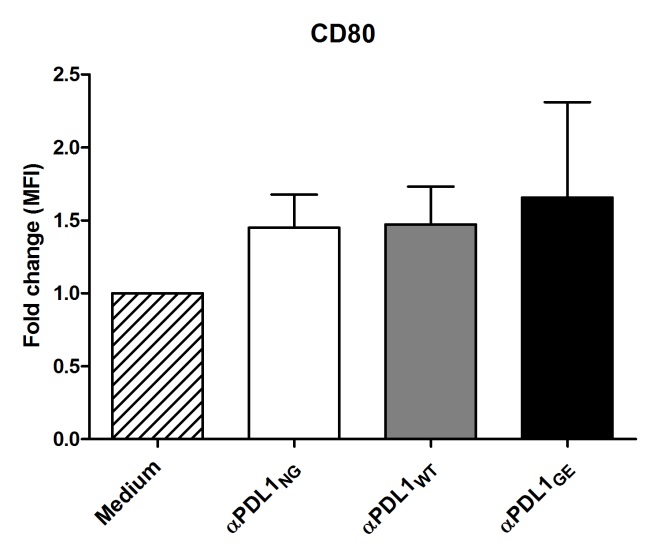

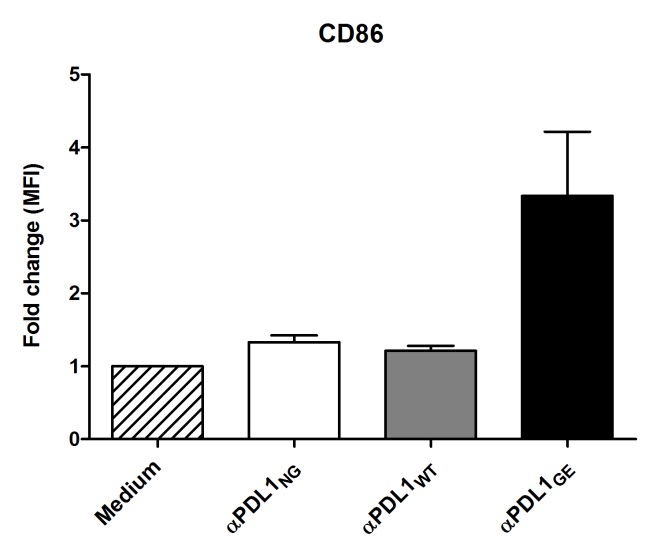
**

**
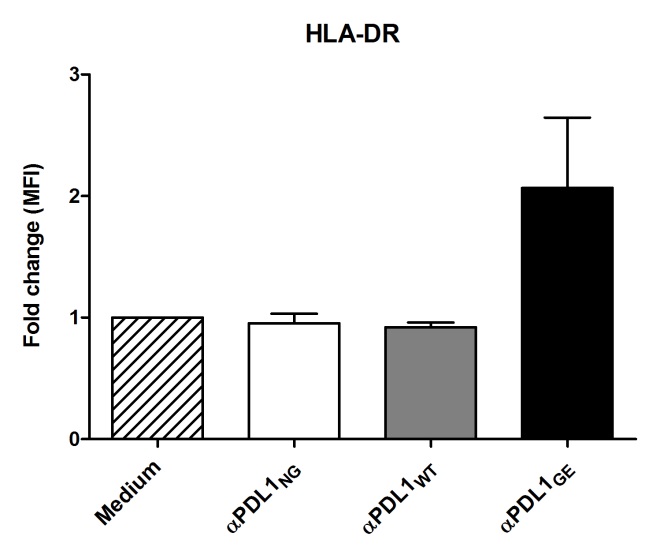
**

**Supplementary Figure 5**: Phenotype of moDCs on day 5 of the MLR. The phenotype of moDC used for the MLR was determined on day 5 of MLR by flow cytometric analysis. MoDC incubated together with T cells (from donor A) and one of the three anti-PD-L1 variants αPDL1_NG_ (white bars), αPDL1_WT_ (gray bars) and αPDL1_GE_ (black bars) or only medium (striped bar) were analyzed. The fold changes in the median fluorescence intensity (MFI) relative to the medium control of CD80^+^, CD86^+^ and HLA-DR^+^ cells in moDC were plotted. Statistics: mean and SEM of four individual experiments were plotted.


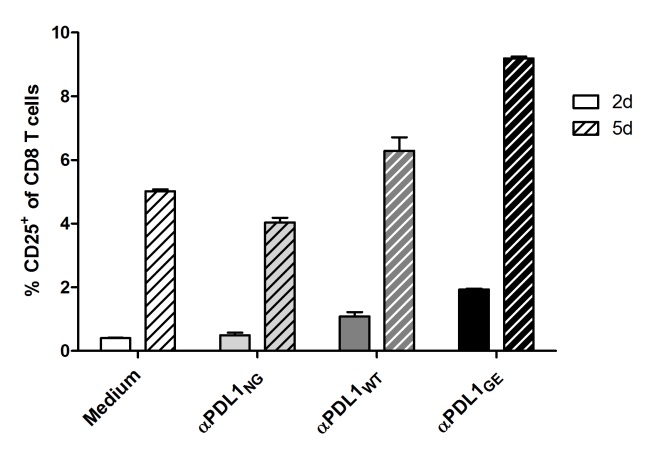

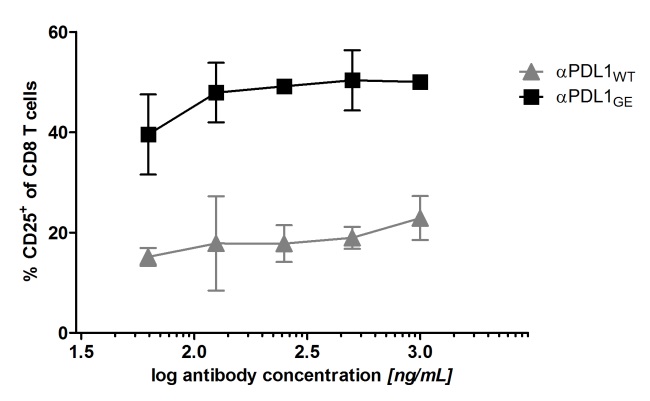


**Supplementary Figure 6**: CD8 T cell activation in a MLR determined on day 2 vs. day 5 and MLR using a concentration series of αPDL1_WT_ and αPDL1_GE_. (6-1) The activation status of CD8 T cells (donor A) in a MLR in absence (white bars) and presence of αPDL1_NG_ (light gray bars), αPDL1_WT_ (dark gray bars), αPDL1_GE_ (black bars) was determined on day 2 (open bars) and 5 (striped bars) by flow cytometric analysis. The relative frequencies of CD25^+^ in CD8 T cells were plotted. (6-2) The activation status of CD8 T cells (donor A) in a MLR in presence of increasing concentrations of αPDL1_WT_ (gray triangles) and αPDL1_GE_ (black squares) was determined on day 5 by flow cytometric analysis. The relative frequencies of CD25+ in CD8 T cells were plotted. Statistics: mean and SD of duplicates were plotted in all graphs. Data using increasing concentrations of anti-PD-L1 antibodies are representative of two independent experiments.


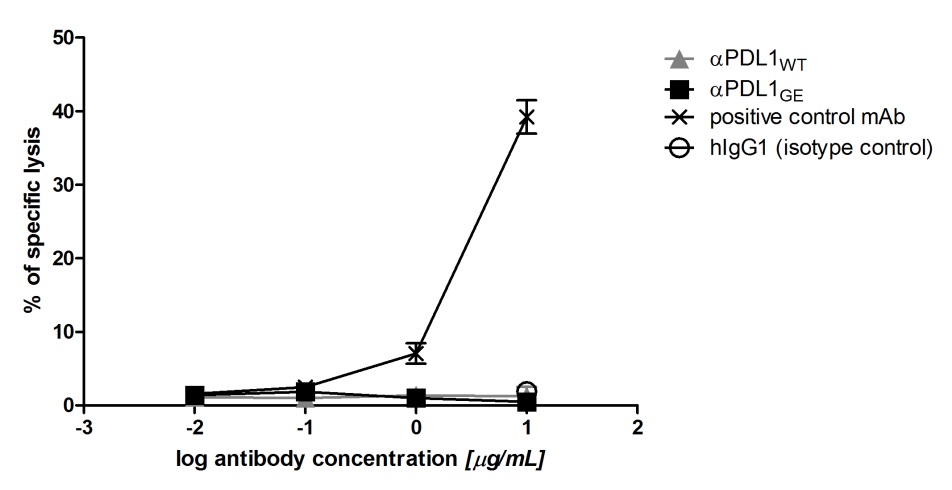


**Supplementary Figure 7:** ADCC assay with ZR-75-1 as target cells. KHYG-1-CD16aV (effectors) were incubated with europium-loaded ZR-75-1 cells (targets) in presence of αPDL1_WT_ (gray triangles), αPDL1_GE_ (black squares), a positive control antibody (black crosses) and a hIgG1 isotype control (white circles) for 5 h with an effector-to-target ratio of 30:1 to determine the specific lysis of target cells. Percentage of specific lysis was plotted against increasing concentrations of test antibody. Statistics: mean and SD of triplicates were plotted. Data are representative of two independent experiments.

**
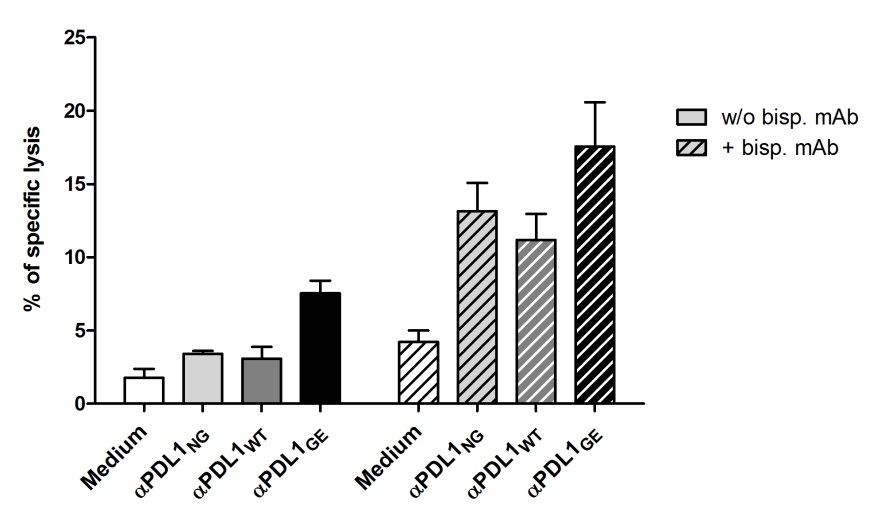
**

**Supplementary Figure 8:** Representative cytotoxicity experiment with pre-activated isolated T cells from donor C pre-activated in a MLR for 5 days in presence of αPDL1_NG_ (light gray bars), αPDL1_WT_ (dark gray bars) and αPDL1_GE_ (black bars). T cells isolated from MLR without addition of test antibody (medium; white bar) served as negative control. Pre-activated T cells were incubated with europium-loaded ZR-75-1 cells for 5 h in ratio of 20:1. The cytotoxicity assay was performed in absence and presence (striped bars) of a bispecific antibody binding to a tumor antigen on ZR-75-1 and to CD3 on T cells. The percentage of specific target cell lysis is shown. Statistics: mean and SEM of triplicates were plotted.
